# A multi-label cascade flexible neural forest model for predicting the subcellular location of multi-site bacterial proteins

**DOI:** 10.1038/s41598-026-58508-9

**Published:** 2026-06-22

**Authors:** Lianxin Zhong, Yang Li, Yanhui Cheng, Guangchang Han, Anqi Li

**Affiliations:** 1 Taishan College of Science and Technology, Tai’an, 271038 China; 2 College of Energy and Mining Engineering, Shandong University of Science and Technology, Qingdao, 266590 China

**Keywords:** Multi-label protein subcellular localization, Cascade forest, Flexible neural tree, Label correlation, Ensemble model, Computational biology and bioinformatics, Mathematics and computing

## Abstract

Predicting the subcellular localization of multi-site bacterial proteins remains challenging because label correlations, limited sample size, and low sequence similarity reduce the effectiveness of conventional multi-label classifiers. In this study, we propose a multi-label cascade flexible neural forest (MLCFN Forest) that combines label-powerset-style coding–classification–decoding with a cascade ensemble of flexible neural tree (FNT) groups. The framework preserves label dependencies, decomposes the induced multi-class task into coordinated binary FNT outputs, and progressively reallocates model capacity to low-confidence samples through confidence-guided sample propagation and feature enhancement across layers. We explicitly note that the cascade FNT backbone builds on our previous CFNForest studies for cancer subtype classification, whereas the present work adapts that backbone to multi-label bacterial protein localization by introducing label-set encoding/decoding, multiclass FNT grouping, and a multi-site protein subcellular localization evaluation workflow. Using Gram-negative and Gram-positive benchmark datasets together with AECA-PSSM and PSSM-DWT features, MLCFN Forest consistently outperformed ML-RBF, ML-KNN, ML-LOC, INSDIF, and MLASSO under jackknife evaluation. For example, it achieved OLA/OAA values of 78.3%/76.4% on the Gram-negative AECA-PSSM dataset, 80.7%/78.5% on the Gram-negative PSSM-DWT dataset, and 80.2%/77.6% on the Gram-positive AECA-PSSM dataset. PCA-based low-dimensional experiments further suggested that the model retained good predictive ability after substantial dimensionality reduction. The present study is limited to classical benchmark datasets and does not yet include an independent external test set, strict nested model selection, or direct benchmarking against protein language model predictors; these points are therefore discussed as limitations and future directions.

## Introduction

Proteins carry out most essential cellular processes, but they can function correctly only when they are transported to the appropriate subcellular compartment. Reliable subcellular localization prediction is therefore important for functional annotation, disease mechanism analysis, drug-target discovery, and proteome-scale screening^1–3^.

Multi-site (multi-label) proteins are of particular interest because a single protein may reside in, or dynamically shuttle between, two or more compartments. Such proteins often participate in transport, signaling, and coordinated metabolic processes, making them biologically informative but also computationally difficult to predict^4–6^.

Experimental determination of protein subcellular localization remains accurate but is labor-intensive, slow, and expensive. As protein sequence databases continue to expand rapidly, computational PSL prediction has become an indispensable complementary strategy in bioinformatics^7,8^.

Early computational PSL studies mainly addressed single-location proteins and relied on traditional classifiers such as k-nearest neighbors, decision trees, neural networks, random forests, hidden Markov models, Bayesian methods, and support vector machines^9–15^. However, these formulations are not designed to preserve label dependencies when proteins occupy multiple locations simultaneously.

To address multi-site proteins, a variety of multi-label predictors have been developed, including ML-KNN, ML-RBF, ML-Loc, InsDif, and MLASSO^16–20^. More recent work has explored label-aware dimensionality reduction, multi-view learning, self-attention, GAN-based architectures, and protein language model representations for multi-label PSL prediction^21–28^. These advances have substantially improved the field, but several challenges remain: preserving higher-order label correlations, adapting model capacity to small and imbalanced bacterial benchmarks, and maintaining performance on proteins with weak sequence similarity.

In addition, prokaryotic localization tools such as PSORTb 3.0 remain important high-precision reference systems for bacterial PSL^29^, while recent protein language model studies have shown that large-scale unsupervised sequence representations can capture structural and functional information relevant to downstream localization tasks^21,22^. These developments raise the bar for new methods and make it necessary to position any proposed model clearly with respect to both classical and modern baselines.

Our previous CFNForest studies^30,31^ introduced cascade flexible neural forest frameworks for cancer subtype classification. The present work does not claim a completely new generic cascade backbone; instead, it adapts that backbone to multi-label bacterial PSL by combining a label-powerset-style transformation^32^ with FNT-based cascade learning. The main contributions of this study are as follows. First, the original multi-label problem is converted into a label-set classification task through coding–classification–decoding so that label correlations are retained as much as possible. Second, an FNT Group is constructed to support multiclass prediction with single-output FNT learners. Third, a confidence-guided cascade mechanism is used to reprocess only difficult samples in deeper layers, thereby reducing unnecessary computation. Fourth, the method is evaluated on benchmark Gram-negative and Gram-positive multi-site bacterial protein datasets using two evolutionary feature representations and a low-dimensional robustness analysis.

## Results

The proposed model was evaluated on Gram-negative and Gram-positive bacterial benchmark datasets. Following common practice for these historical multi-site PSL benchmarks, we used the jackknife (leave-one-out cross-validation) protocol^33–35^ and reported overall localization accuracy (OLA) and overall actual accuracy (OAA) as the primary endpoints, because these are the metrics consistently reported by the compared methods on the same datasets^16–20,36^. We now state explicitly that jackknife is a cross-validation procedure, and we further discuss the absence of class-wise F-scores and ROC/AUC curves as a limitation of the current benchmark setting rather than as an omitted claim of superiority.

### Model Testing on Gram-negative bacteria dataset

#### AECA-PSSM for Gram-negative bacteria dataset

We first compared MLCFN Forest with five representative multi-label learning models introduced in the Introduction section—ML-KNN^16^, ML-RBF^17^, ML-LOC^18^, INSDIF^19^, and MLASSO^20^—on the Gram-negative bacterial dataset using AECA-PSSM features. The corresponding results are listed in Table 1.

**Table 1:** Prediction results for protein subcellular localization on the Gram-negative dataset using different models.

Datasets	Performance	Jackknife Test (%)					
		Algorithms					
		ML-RBF	ML-KNN	ML-LOC	INSDIF	MLASSO	MLCFN Forest
Gram- negative	OLA	62.4	66.3	64.4	65.0	60.6	78.3
	OAA	59.7	64.1	64.4	62.7	61.1	76.4

OLA and OAA were used to evaluate model performance. OLA measures the proportion of correctly predicted localization assignments among all localized proteins, whereas OAA is a stricter metric that counts a sample as correct only when its entire label set is predicted correctly. In multi-site PSL, OAA is particularly important because it directly evaluates whether all locations of a protein are recovered simultaneously.

$${\rm{OLA = }}\frac{{\rm{1}}}{{\mathop \sum \nolimits_{{\rm{i = 1}}}^{\rm{N}} {\rm{|L(}}{{\rm{Q}}_{\rm{i}}}{\rm{)|}}}}\mathop \sum \limits_{{\rm{i = 1}}}^{\rm{N}} {\rm{|M(}}{{\rm{Q}}_{\rm{i}}}{\rm{)}} \cap {\rm{L(}}{{\rm{Q}}_{\rm{i}}}{\rm{)|}}$$

where $\:{\mathcal{M}(}{\mathrm{Q}}_{\mathrm{i}}\mathrm{)}$ is the subcellular site of the protein sequence $\:{\mathrm{Q}}_{\mathrm{i}}$ predicted by the model, and $\:{\mathcal{L}(}{\mathrm{Q}}_{\mathrm{i}}\mathrm{)}$ is its actual site.

Because OAA requires an exact match of the full label set, it is generally more difficult to optimize than OLA and therefore provides a conservative indicator of real multi-label performance. For this reason, we interpret OAA as the primary practical indicator while still reporting both metrics for completeness.

$$\:\mathrm{OAA=}\frac{\mathrm{1}}{\mathrm{N}}\sum\:_{\mathrm{i=1}}^{\mathrm{N}}{|\Delta}{[M(}{\mathrm{Q}}_{\mathrm{i}}\mathrm{),L(}{\mathrm{Q}}_{\mathrm{i}}\mathrm{)]}$$

and,

$$\Delta\:{\rm{[M(}}{{\rm{Q}}_{\rm{i}}}{\rm{),L(}}{{\rm{Q}}_{\rm{i}}}{\rm{)] = \{ }}\begin{array}{*{20}{c}} {{\rm{1,}}}&{{\rm{~if~~M(}}{{\rm{Q}}_{\rm{i}}}{\rm{) = L(}}{{\rm{Q}}_{\rm{i}}}{\rm{)}}}\\ {{\rm{0,}}}&{{\rm{~otherwise~}}} \end{array}$$

Table 1 shows that MLCFN Forest achieved the best performance among all compared models on both OLA and OAA. In particular, the OAA of MLCFN Forest reached 76.4%, indicating that the proposed label-set preserving cascade model can effectively identify complete localization combinations on the Gram-negative AECA-PSSM benchmark.

## PSSM-DWT for gram-negative bacteria dataset

To further evaluate robustness with respect to feature representation, we repeated the Gram-negative experiments using PSSM-DWT features and compared MLCFN Forest against the same five multi-label baselines. The ML-KNN baseline was run with k = 1, following the setting used in the original experiments for this manuscript. And we would like to clarify that retaining this setting is intended to ensure direct comparability on these small benchmark datasets, and does not imply that this choice represents a universally optimal k value.

**Fig. 1:**
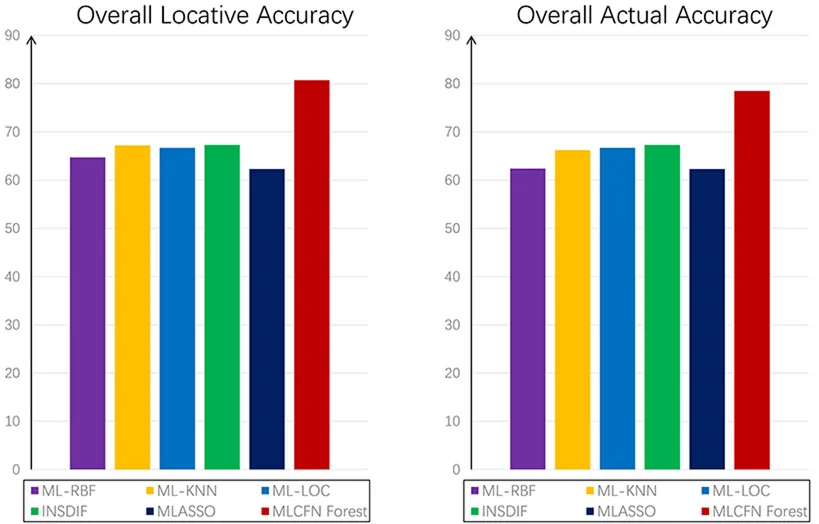
Prediction results for protein subcellular localization on the Gram-negative dataset using different models.

Figure 1 shows that the proposed method again produced the highest OLA and OAA. On the Gram-negative PSSM-DWT dataset, MLCFN Forest reached 80.7% OLA and 78.5% OAA, outperforming the other multi-label baselines and suggesting that the cascade FNT framework remains effective across different evolutionary feature encodings.

## Model Testing on Gram-positive bacteria dataset

### AECA-PSSM for Gram-positive bacteria dataset

We next evaluated MLCFN Forest on the Gram-positive bacterial dataset using AECA-PSSM features and compared it with ML-RBF, ML-KNN, ML-LOC, INSDIF, and MLASSO. The results are summarized in Table 2.

**Table 2:** Prediction results of Gram-positive dataset protein subcellular localization in different models.

Datasets	Performance	Jackknife Test (%)					
		Algorithms					
		ML-RBF	ML-KNN	ML-LOC	INSDIF	MLASSO	MLCFN Forest
Gram- positive	OLA	65.4	69.0	68.3	68.8	66.3	80.2
	OAA	65.5	68.3	67.9	67.9	65.9	77.6

Table 2 indicates that the proposed model also performed best on the Gram-positive AECA-PSSM benchmark. The OAA of MLCFN Forest reached 77.6%, showing that the method generalizes well to the smaller four-location Gram-positive dataset.

### PSSM-DWT for Gram-positive bacteria dataset

**Fig. 2:**
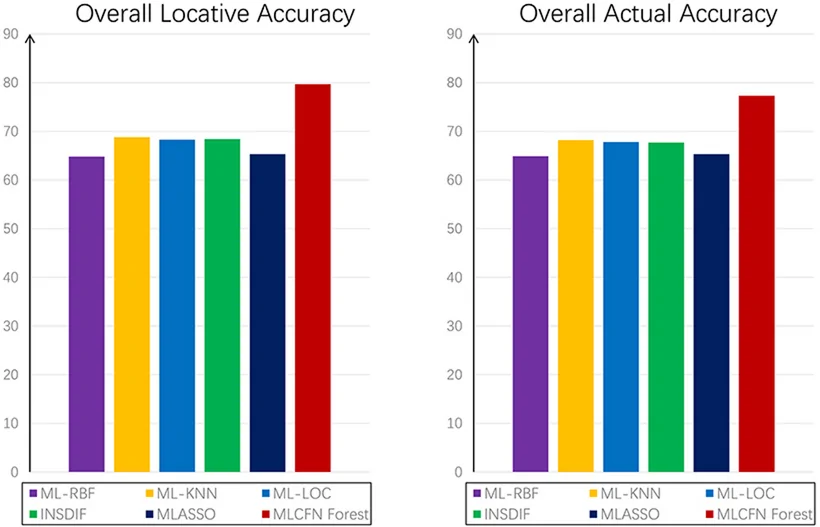
Prediction results for protein subcellular localization on the Gram-positive dataset using different models.

To examine the influence of an alternative feature representation on the Gram-positive benchmark, we also extracted PSSM-DWT features and compared MLCFN Forest with the same five baselines. The results are shown in Fig. 2.

The left panel of Fig. 2 compares OLA across models, whereas the right panel compares OAA. MLCFN Forest again showed the best overall behavior, achieving an OAA of 77.3% on this dataset. Taken together, the Gram-negative and Gram-positive results suggest that the proposed method is stable across both bacterial groups and across both evolutionary feature extraction pipelines.

## Effect of dimension reduction on results

Dimensionality reduction is an important issue for multi-site protein PSL because high-dimensional sequence descriptors often contain correlated and redundant components. To test whether the proposed model remains effective under a heavily compressed representation, we applied principal component analysis (PCA) after feature extraction and evaluated MLCFN Forest in a low-dimensional sample space.

**Fig. 3:**
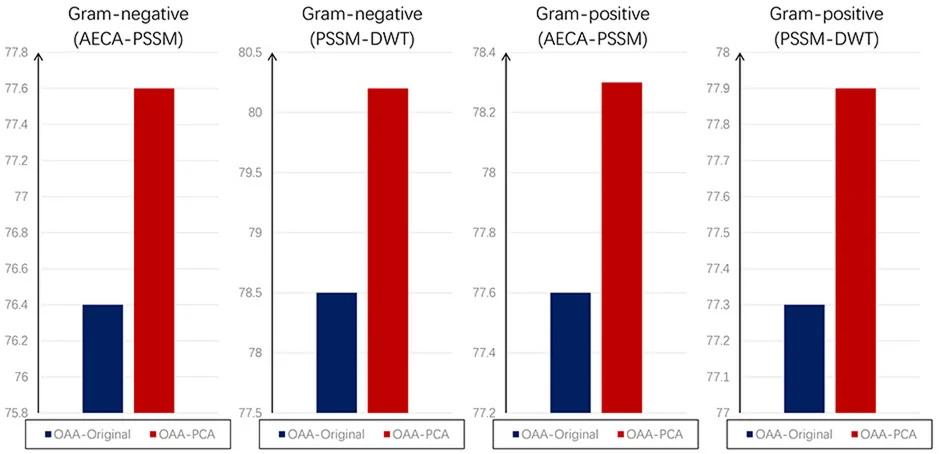
Overall actual accuracy after principal component analysis on the Gram-positive and Gram-negative bacterial datasets.

Two feature extraction methods were used to encode the protein sequences. After obtaining the corresponding feature matrices, PCA was applied and the feature dimension was reduced to seven. We now clarify that this seven-dimensional setting was used as a stress-test of low-dimensional robustness rather than as a claim of optimal dimensionality selected by an explained-variance threshold. The resulting low-dimensional experiments are shown in Fig. 3.

As shown in Fig. 3, PCA-based compression improved OAA to varying degrees relative to the original high-dimensional features, suggesting that part of the original variance was redundant or noisy for this task. Importantly, MLCFN Forest maintained strong performance even after aggressive dimensionality reduction, which supports the idea that the model can still extract useful decision structure in a compact feature space. At the same time, we do not over-interpret the biological meaning of the seven principal components, and we now identify more interpretable explainable-AI analyses as future work.

## Discussion

The experimental results suggest that MLCFN Forest benefits from three mutually reinforcing design choices. First, the coding–classification–decoding strategy preserves label-set information instead of predicting each label independently. Second, the adaptive FNT backbone can evolve task-specific structures during training, which is attractive for small bacterial PSL datasets. Third, the confidence-guided cascade reallocates later-layer capacity to ambiguous proteins rather than repeatedly processing all samples, which helps the model focus on hard cases.The current study extends that line of work to multi-label bacterial PSL by introducing label-set transformation, multiclass FNT grouping, and evaluation on classical Gram-positive and Gram-negative multi-site protein benchmarks.

Several limitations should also be emphasized. The study relies on classical benchmark datasets and jackknife evaluation rather than an external independent test set. Hyperparameter tuning was not performed in a fully nested validation framework. In addition, although the original benchmark datasets were constructed under redundancy control, PSI-BLAST-based PSSM generation against an external database may still introduce homolog-related information that cannot be fully excluded. Finally, direct benchmarking against PSORTb 3.0^29^, protein language model predictors such as DeepLoc 2.0^22^, and class-wise metrics such as micro/macro-F1 or ROC-AUC was beyond the scope of the current experiments. We now state these points explicitly so that the strengths and limits of the current work are presented more transparently.

## Conclusion

In this study, we investigated the prediction of multi-site bacterial protein subcellular localization using a multi-label cascade flexible neural forest. By combining a label-powerset-style coding–classification–decoding strategy with adaptive FNT-based cascade learning, the model preserved label dependencies and achieved consistently improved OLA/OAA values on Gram-negative and Gram-positive benchmark datasets. In future work, we will focus on more rigorous homology-partitioned evaluation, external validation, class-wise metrics, direct comparisons with PSORTb and protein language model baselines, as well as more interpretable feature attribution analyses.

### Methods

#### Datasets

To evaluate MLCFN Forest on established multi-site bacterial PSL benchmarks, we used the Gram-positive dataset reported by Shen and Chou^37^ and the Gram-negative dataset reported by Chou and Shen^38^. These benchmarks are classical datasets in the bacterial multi-location PSL literature and are typically evaluated by jackknife rather than by a pre-fixed train/test split. The Gram-positive dataset contains 519 different protein sequences distributed across four subcellular locations and 523 locative proteins in total, whereas the Gram-negative dataset contains 1392 different protein sequences distributed across eight locations and 1456 locative proteins in total.

### Validation protocol, redundancy control, and leakage considerations

The jackknife protocol leaves one protein out as the test sample in each iteration and uses the remaining *N* − 1 proteins for training. In this study, the same model configuration was maintained throughout the jackknife iterations, and the held-out labels were not used during classifier fitting.Meanwhile, to facilitate fair comparison with previously published results, we did not adopt a fully nested hyperparameter optimization procedure; instead, we followed the standard jackknife protocol commonly used for these benchmark datasets^33–35^.

The original Gram-positive and Gram-negative benchmark datasets were constructed under stringent redundancy control, with no pairwise sequence identity of 25% or higher within the same localization subset^37,38^. This reduces homology bias at the benchmark level. However, because PSSM profiles are generated by PSI-BLAST against an external sequence database, homolog-related information leakage through that database cannot be completely ruled out. In future work, we will continue to make efforts to overcome this limitation Table 3.

**Table 3:** Breakdown of the Gram-negative and Gram-positive bacterial benchmark datasets.

Datasets	Subset	Subcellular location name	Number of proteins
Gram- negative	S1	Cell inner membrane	557
	S2	Cell outer membrane	124
	S3	Cytoplasm	410
	S4	Extracellular	133
	S5	Fimbrium	32
	S6	Flagellum	12
	S7	Nucleoid	8
	S8	Periplasm	180
	NO. locative proteins	1456	
	NO. different proteins	1392	
Gram- positive	S1	Cell membrane	174
	S2	Cell wall	18
	S3	Cytoplasm	208
	S4	Extracellular	123
	NO. locative proteins	523	
	NO. different proteins	519	

### Feature extraction method

Machine learning models operate on numerical vectors rather than on raw amino-acid sequences. Therefore, each protein sequence must first be encoded into a feature vector that captures relevant biological information. In this study, we used two PSSM-derived evolutionary descriptors for multi-site protein prediction: a 190-dimensional absolute entropy correlation analysis representation (AECA-PSSM) and a 480-dimensional discrete wavelet transform representation (PSSM-DWT)^36^.

### AECA-PSSM

A position-specific scoring matrix (PSSM), typically generated by PSI-BLAST, represents evolutionary information for a protein sequence^39,40^. Following the standard settings used in previous PSSM-based studies by our group and related work^36,41^, PSI-BLAST was run against the non-redundant (NR) database with an E-value threshold of 0.001 for three iterations to produce the PSSM for each protein sequence.

$$\:\mathrm{P}\mathrm{S}\mathrm{S}\mathrm{M}=\left[\begin{array}{cccc}{\mathrm{N}}_{1\to\:1}&\:{\mathrm{N}}_{1\to\:2}&\:\cdots\:&\:{\mathrm{N}}_{1\to\:20}\\\:{\mathrm{N}}_{2\to\:1}&\:{\mathrm{N}}_{2\to\:2}&\:\cdots\:&\:{\mathrm{N}}_{2\to\:20}\\\:\vdots&\:\vdots&\:\vdots&\:\vdots\\\:{\mathrm{N}}_{\mathrm{i}\to\:1}&\:{\mathrm{N}}_{\mathrm{i}\to\:2}&\:\cdots\:&\:{\mathrm{N}}_{\mathrm{i}\to\:20}\\\:\vdots&\:\vdots&\vdots&\:\vdots\\\:{\mathrm{N}}_{\mathrm{L}\to\:1}&\:{\mathrm{N}}_{\mathrm{L}\to\:2}&\:\cdots\:&\:{\mathrm{N}}_{\mathrm{L}\to\:20}\end{array}\right]$$

Where $\:\mathrm{L}$ is the number of amino acid residues in the protein sequence. $\:{\mathrm{N}}_{\mathrm{i}\to\:\mathrm{j}}$ describes the relative probability describing how the $\:\mathrm{i}$ th amino acid position in the protein sequence mutates into the $\:\mathrm{j}$ th amino acid type during biological evolution processes. After obtaining the PSSM of a given protein, the element $\:{\mathrm{N}}_{\mathrm{i}\to\:\mathrm{j}}$ can be normalized by:

$$\:{\mathrm{P}}_{\mathrm{i}\mathrm{j}}=\frac{1}{1+{\mathrm{e}}^{-{\mathrm{N}}_{\mathrm{i}\to\:\mathrm{j}}}}$$

After normalization, each column of the PSSM can be interpreted as a probability distribution over amino-acid substitutions. AECA-PSSM quantifies the pairwise relationships among these 20 column-wise distributions by means of absolute entropy correlation analysis, thereby producing a compact 190-dimensional descriptor that retains evolutionary dependence information while reducing redundancy compared with a full asymmetric relative-entropy encoding.

Relative entropy (also known as Kullback-Leibler divergence)^42^ describes the difference between two probability distributions P and Q, and it is a measure of asymmetry. If the relative entropy is directly used to describe the relationship between each two columns in PSSM, a 19 × 20 = 380-dimensional feature vector will be needed to fully extract the information in the PSSM. Absolute entropy is a modified form of relative entropy. The absolute entropy between two different probability distributions P and Q is as follows:

$$\begin{array}{*{20}{c}} {{\rm{D(P,Q)}}}&{{\rm{ = }}\frac{{\rm{1}}}{{\rm{2}}}{\rm{(}}{{\rm{D}}_{{\rm{KL}}}}{\rm{(P}}\parallel {\rm{(Q) + }}{{\rm{D}}_{{\rm{KL}}}}{\rm{(P}}\parallel {\rm{Q))}}}\\ {}&{{\rm{ = }}\frac{{\rm{1}}}{{\rm{2}}}\mathop \sum \limits_{{\rm{i = 1}}}^{\rm{N}} {\rm{(P(i) - Q(i))log(}}\frac{{{\rm{P(i)}}}}{{{\rm{Q(i)}}}}{\rm{)}}} \end{array}$$

Where $\:{\mathrm{D}}_{\mathrm{K}\mathrm{L}}$ is the relative entropy of P and Q, defined as:

$$\:\begin{array}{cc}{\mathrm{D}}_{\mathrm{K}\mathrm{L}}(\mathrm{P}\parallel\:\mathrm{Q})&\:=\sum\:_{\mathrm{i}=1}^{\mathrm{N}}\mathrm{P}\left(\mathrm{i}\right)\mathrm{l}\mathrm{o}\mathrm{g}\left(\frac{1}{\mathrm{Q}\left(\mathrm{i}\right)}\right)-\sum\:_{\mathrm{i}=1}^{\mathrm{N}}\mathrm{P}\left(\mathrm{i}\right)\mathrm{l}\mathrm{o}\mathrm{g}\left(\frac{1}{\mathrm{P}\left(\mathrm{i}\right)}\right)\\\:&\:=\sum\:_{\mathrm{i}=1}^{\mathrm{N}}\mathrm{P}\left(\mathrm{i}\right)\mathrm{l}\mathrm{o}\mathrm{g}\left(\frac{\mathrm{P}\left(\mathrm{i}\right)}{\mathrm{Q}\left(\mathrm{i}\right)}\right)\:\end{array}$$

For the PSSM to be considered as a 20-dimensional probability distribution, absolute entropy correlation analysis was used to analyze the pairwise relationships between every two columns in the PSSM and to extract protein sequence information. A 190-dimensional feature was created by absolute entropy correlation analysis. Compared with using relative entropy, the size of the feature was reduced by half and the calculation was simpler.

The absolute entropy is always equal to or greater than zero, and when it is zero it indicates that the two distributions are identical. Since the lengths of different protein sequences are not the same, the final calculation is averaged by dividing the sequence lengths into the effects of eliminating protein lengths.

### PSSM-DWT

**Fig. 4:**
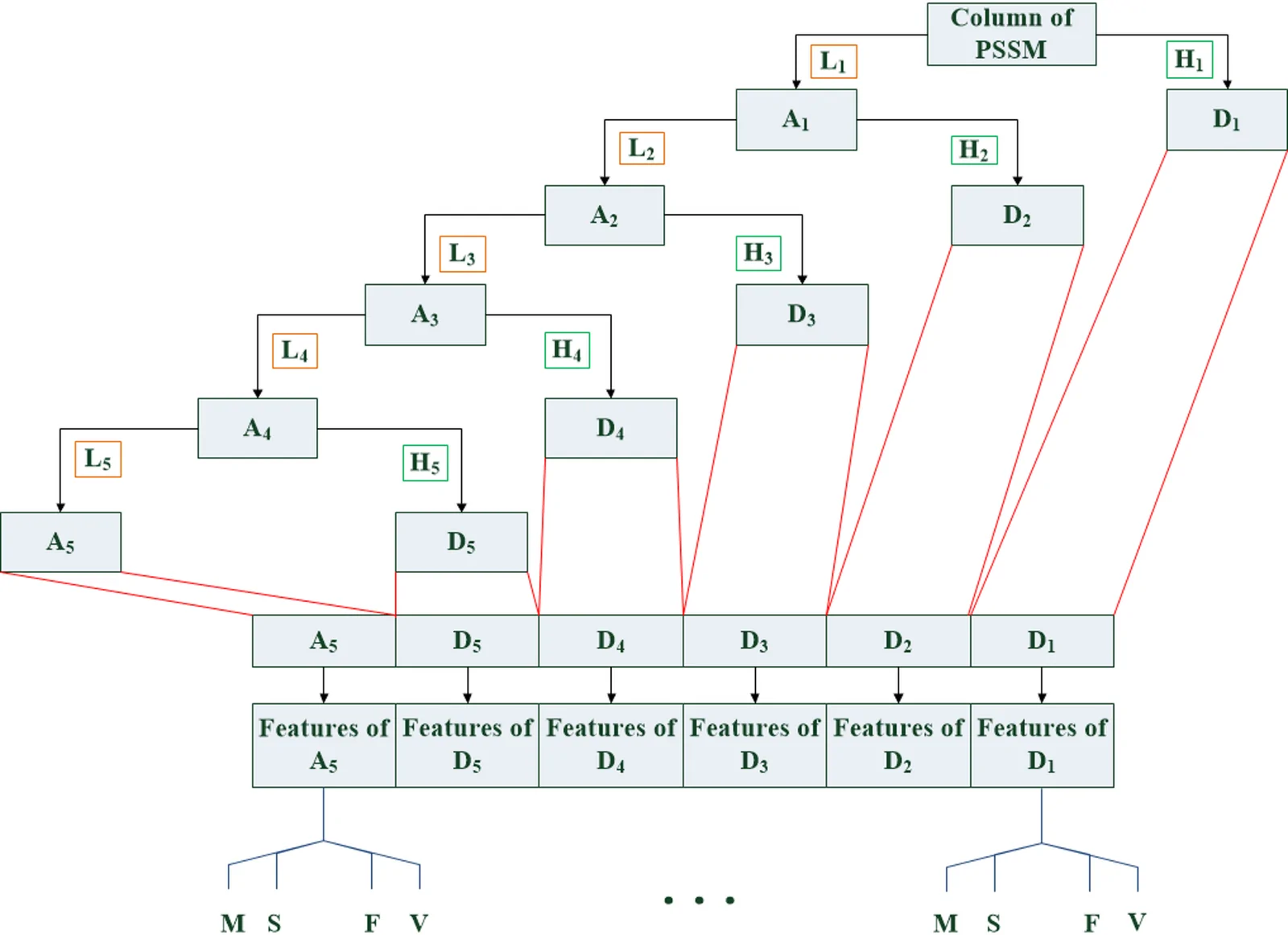
Process of PSSM-DWT feature extraction; the upper branch denotes high-pass filtering and the lower branch denotes low-pass filtering.

The discrete wavelet transform (DWT) is a signal-processing tool that decomposes a signal into multi-resolution approximation and detail components. When applied column-wise to a PSSM, it captures both local fluctuation patterns and coarser distributional structure^43^. In this study, a five-level DWT was applied to each PSSM column to obtain approximation coefficients (A5) and detail coefficients (D1–D5) Fig. 4.

For each sub-band, four statistics were extracted to describe the time–frequency distribution of the transformed signal: M (mean absolute coefficient), S (standard deviation), F (fluctuation index), and V (coefficient of variation of the absolute coefficients). These quantities summarize both average magnitude and variability across the wavelet sub-bands.

$${\rm{F = }}\frac{{\rm{1}}}{{\rm{N}}}\mathop \sum \limits_{{\rm{t = 1}}}^{{\rm{N - 1}}} {\rm{|c(t + 1) - c(t)|}}$$

$${\rm V}=\frac{\sigma}{\upmu}=\frac{{\frac{{\rm{1}}}{{\rm{N}}}\sum_{{\rm{t = 1}}}^{\rm{N}} {\rm{|c(t)|}}}}{{\sqrt {\frac{{\rm{1}}}{{\rm{N}}}\mathop \sum _{{\rm{t = 1}}}^{\rm{N}} {{{\rm{(|c(t)|-\mu )}}}^{\rm{2}}}}}}$$

Feature M summarizes the average magnitude distribution of each sub-band, whereas S, F, and V characterize dispersion and fluctuation. Because 24 statistics are obtained from each of the 20 PSSM columns, the final PSSM-DWT descriptor is 480-dimensional. More detailed derivations of both feature extraction procedures are available in^36^.

### Principal component analysis for dimensionality reduction

When building a protein feature representation, it is desirable to retain as much relevant sequence information as possible; however, highly correlated variables may also introduce noise, instability, and unnecessary computational burden. For this reason, dimensionality reduction was explored as an auxiliary robustness analysis rather than as the primary production setting of the predictor.

Principal component analysis (PCA) transforms the original features into mutually orthogonal components ordered by variance contribution^44^. In this manuscript, PCA was used to test whether the proposed classifier remained effective after aggressive compression to seven dimensions. It should be noted that the choice of seven components was empirical and intended as a low-dimensional stress-test; it was not selected via a formal explained-variance threshold, nor is it presented as the only biologically meaningful representation. More interpretable alternatives, including label-aware dimensionality reduction and SHAP-style explanation methods, are valuable future directions.

### Multi-label cascade flexible neural forest model

The algorithmic design strongly influences the final prediction performance in multi-site protein localization. In this study, we propose a multi-label cascade flexible neural forest (MLCFN Forest) that adapts the previously published CFNForest backbone^30,31^ to a multi-label bacterial PSL setting.

### Flexible neural tree

**Fig. 5:**
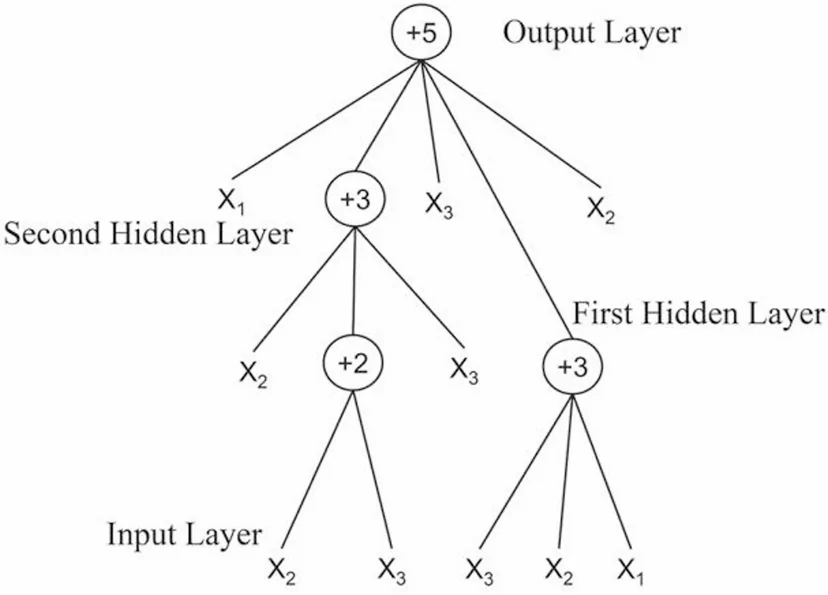
A typical representation of the flexible neural tree model.

A flexible neural tree (FNT) can adaptively optimize both its structure and parameters during training, while allowing cross-layer information flow between nodes^32–35^. Because the tree topology is evolved rather than fixed in advance, FNTs are attractive when the appropriate network structure is not known a priori, as is often the case for small biological datasets Fig. 5.

To construct an FNT, we define a function set F and a terminal set T. The terminal set contains the input features, whereas + n in the function set denotes a computation node with n child inputs. Different choices of F yield structurally different trees and therefore increase model diversity. In this study, the logistic function was used as the activation function of the FNT.

The output of an FNT is computed recursively by a depth-first traversal of the tree. For a non-leaf computation node with n inputs, the neuron output is obtained by applying the activation function to the weighted sum of its child outputs, i.e., sigma(sum_{j = 1}^{n} w_j x_j), where x_j denotes the j-th input and w_j its associated weight. In this way, each non-leaf node acts as a small adaptive neuron whose inputs may come either from terminal features or from lower-level function nodes.

$$\:\sum\:_{\mathrm{n}}=\sum\:_{\mathrm{j}=1}^{\mathrm{n}}{\mathrm{w}}_{\mathrm{j}}\ast\:{\mathrm{x}}_{\mathrm{j}}$$

Here, w_j denotes the connection weight and x_j denotes the corresponding input. The construction of an FNT therefore involves two coupled optimization steps: tree-structure evolution and parameter estimation.

**Fig. 6:**
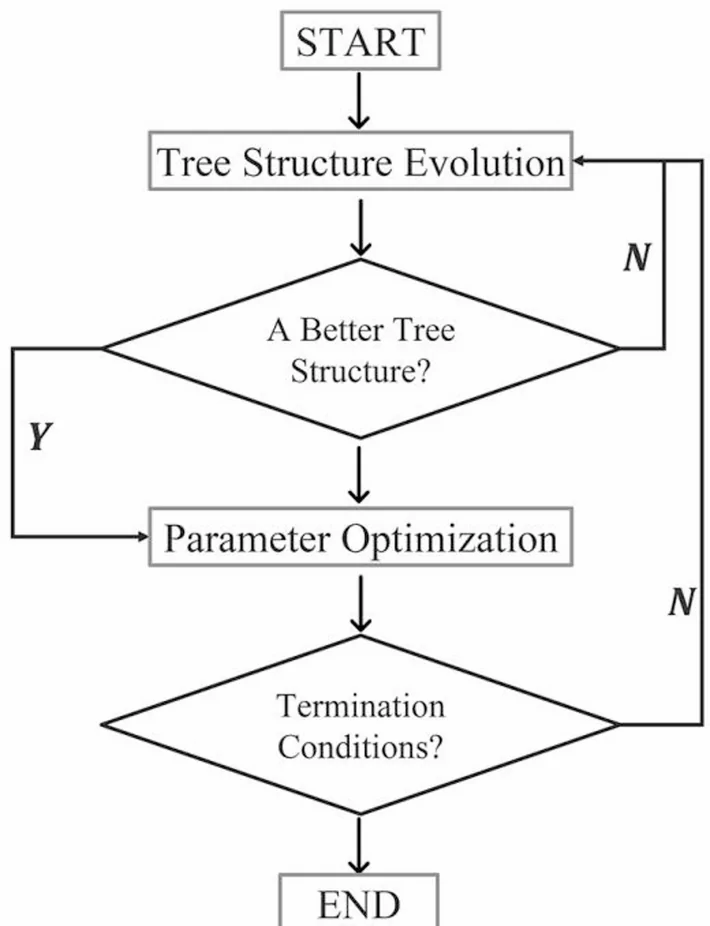
Pipeline of the flexible neural tree model, adapted from our previous CFNForest studies^37,46]^. The algorithm stops when the termination conditions are met.

Figure 6 summarizes the FNT optimization pipeline. As in our previous CFNForest implementation^37,46]^, grammar-guided genetic programming is used for structure evolution and particle swarm optimization (PSO) is used for parameter tuning. To improve reproducibility, we now clarify that the implementation follows the same parameter configuration reported in those studies, including the grammar-guided evolutionary search and the PSO-based parameter refinement. Because the final FNT topology is data-adaptive, the model does not have a single fixed parameter count in the same sense as a conventional dense neural network.

### Implementation and hyperparameter settings

The maximum cascade depth was set to four following our previous CFNForest studies^37,46]^, which provided a practical upper bound that avoids premature stopping while limiting unnecessary structural growth. The confidence interval used for sample screening is illustrated in Fig. 8. Across layers, different function sets were used to increase structural diversity. We now describe these settings explicitly because they affect reproducibility even though the internal FNT topology is optimized automatically during training.

### Multi-label learning

Let X denote the d-dimensional input space and Y = {lambda_1, …, lambda_q} the set of possible subcellular labels. Each training sample consists of a feature vector x in X and a subset of relevant labels Y_x contained in Y. The goal of multi-label learning is to learn a function h: X -> 2^Y that predicts the correct label subset for an unseen protein.

The main difficulty of multi-label learning lies in the explosive growth of the output space and in the need to model label dependence. For protein PSL, this dependence is biologically meaningful because a protein that localizes to one compartment may have strong tendencies to co-localize with specific additional compartments.

From the perspective of label-correlation modeling, multi-label algorithms are often categorized as first-order, second-order, or higher-order strategies. First-order approaches decompose the task into independent binary problems and therefore ignore label dependence^38–40^. Second-order methods model pairwise correlations^40–42^. Higher-order approaches treat the label set more holistically and are better suited when the full label combination itself is informative^43,44^.

Because subcellular localization labels of the same protein are often strongly correlated, we use a label-powerset-style transformation [48] to map each observed label subset to a unique class identifier. In other words, the full localization set of a protein is encoded as a single multiclass target and decoded after prediction. This preserves observed label correlations, but it also implies that unseen label combinations are difficult to recover; we now discuss that trade-off explicitly as a limitation of the formulation.

#### Multi-label cascade flexible neural forest model

Although FNT is a flexible and expressive learner, its native single-output structure is not directly suited to multi-class or multi-label classification. To address this, we construct MLCFN Forest by combining label-set encoding with an ensemble of coordinated FNT learners.

**Fig. 7:**
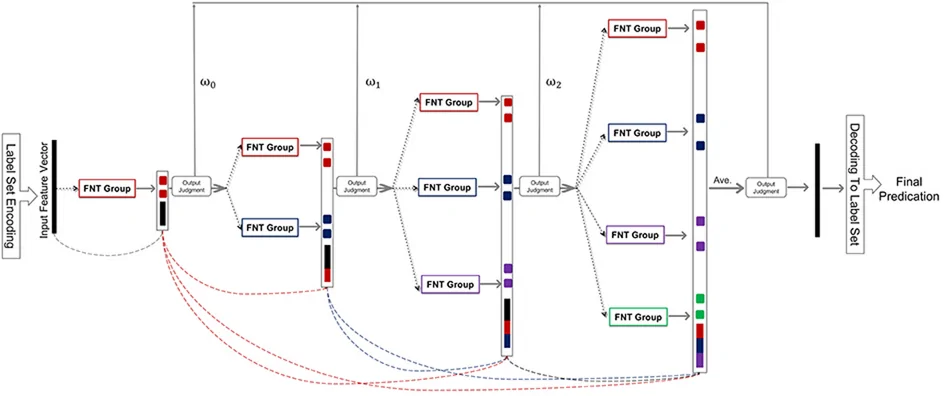
The multi-label cascade flexible neural forest model.

First, the observed label subsets are encoded into class identifiers. All proteins sharing the same localization combination are assigned to the same class. Once the correct class is predicted, the original localization set can be recovered by decoding. This design preserves the observed label combination as a whole instead of predicting each location independently.

To solve the induced multiclass problem with single-output FNTs, we introduce the concept of an FNT Group. The multiclass target is represented by an M-ary code, and each bit of the code is learned by one FNT. Consequently, an M-class problem requires ceil(log2 M) coordinated FNTs in one group. For example, the four-class Gram-positive problem requires two FNTs per group, whereas the eight-class Gram-negative problem requires three. The outputs of the constituent FNTs are then combined and decoded to recover the predicted class.

Within each FNT Group, the constituent trees are trained under a standard bagging scheme: for each code bit, an FNT is fitted on a bootstrap resample of the training data available at that layer (using a resample size equal to the layer-specific training-set size). The bitwise outputs are averaged to obtain a group-level confidence vector, which is then decoded into the predicted class. This clarification is added to make the ensemble procedure more reproducible.

**Fig. 8:**
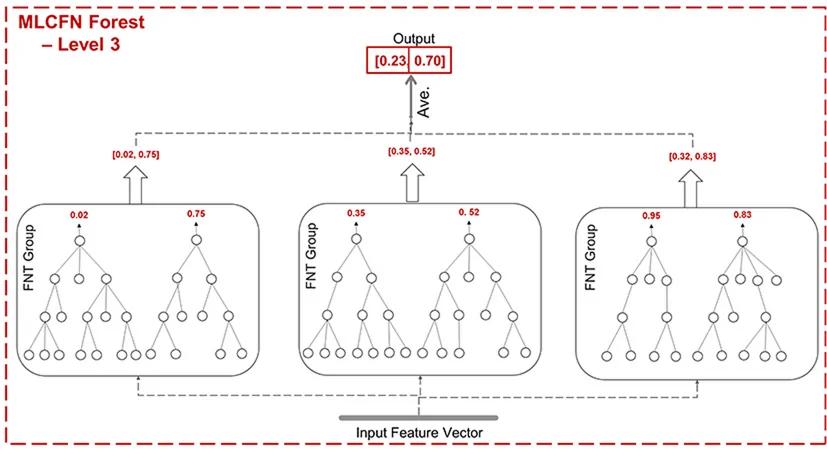
Structure of the third cascade layer when each FNT Group contains two FNTs. If the confidence interval is set to [0, 0.2] U [0.8, 1], samples outside the interval are assigned to the low-confidence set X and are reprocessed in the next layer.

The first cascade layer classifies all samples. After this initial prediction, a confidence-based screening mechanism divides the samples into a high-confidence set Y and a low-confidence set X. Proteins in Y are not forwarded to deeper layers, whereas proteins in X are reclassified by later layers. This mechanism reduces repeated computation on already well-classified samples and concentrates model capacity on difficult cases.

To increase structural diversity across the cascade, we introduce an FNT Group with a different function set at each subsequent layer. In this study, the four forests use the function sets {+2, + 3, +4}, {+2, + 3, +5}, {+2, + 4, +5}, and {+3, + 4, +5}, respectively. This layer-by-layer widening strategy follows the idea that deeper layers should provide additional representational diversity without unnecessarily expanding the model for easy samples.

**Fig. 9:**
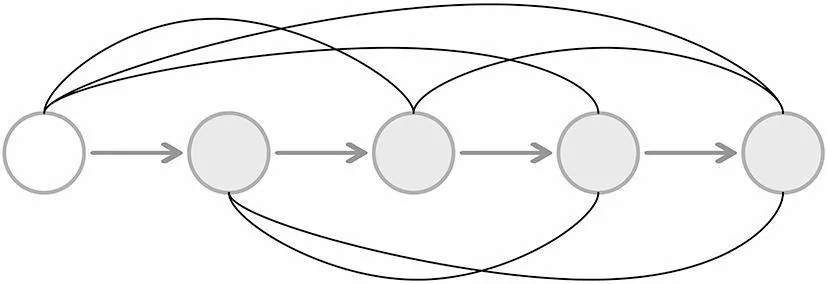
Dense-block style feature propagation concept adapted from DenseNet [^45^].

Because multi-site bacterial PSL datasets are relatively small, we further augment the representation by passing the enhanced feature outputs of earlier layers to later layers. These enhanced feature points are additional learned descriptors generated inside the cascade and concatenated with the original features before the next layer is trained Fig. 9.

The propagation of enhanced feature points is inspired by the dense connectivity idea of DenseNet [59], where outputs from previous layers are reused as inputs to subsequent layers. In the present model, this mechanism is used conceptually rather than as a literal CNN block: the newly generated layer-wise features are concatenated with earlier representations so that later FNT Groups receive richer input information, which is particularly useful on small-sample datasets.

To reduce the risk that a single cascade layer dominates the final decision, the outputs of all layers are combined by a weighted aggregation rule. Deeper layers receive larger weights because they process the harder low-confidence samples, but earlier layers still contribute to the final prediction so that useful information is not discarded.

### Computational considerations

MLCFN Forest is not characterized by a single fixed parameter count because each FNT is evolved adaptively. If L denotes the number of cascade layers, B_l the number of FNTs in layer l, T_l the average number of non-leaf nodes in an FNT, and n_l the number of samples entering layer l, then the effective training cost grows with the cumulative structure-search and parameter-optimization cost over all layers, while the per-sample inference cost is proportional to sum_l B_l T_l. In practice, the sample-screening mechanism reduces n_l in deeper layers, which lowers the effective computational burden relative to forwarding all samples through all layers. We now include this theoretical discussion because hardware-specific runtime and memory benchmarks were not available for the current revision.

$$\:{\mathrm{y}}_{\mathrm{f}}=\sum\:_{\mathrm{i}=1}^{\mathrm{N}}{{\upomega\:}}_{\mathrm{i}}\ast\:{\mathrm{y}}_{\mathrm{i}}$$ ($$\:{{\upomega\:}}_{\mathrm{i}}=\mathrm{i}/1+2+\dots\:+\mathrm{i}$$).

In the above aggregation rule, N denotes the number of cascade layers, y_i denotes the output of the i-th layer, and omega_i denotes the corresponding contribution weight.

After the final class predictions are obtained, the encoded class identifiers are decoded back into the corresponding localization label sets. The resulting predictions are then evaluated using OLA and OAA to assess the performance of the proposed model on multi-site protein subcellular localization.

## Data Availability

The Gram-positive and Gram-negative benchmark datasets used in this study are available from the original publications [37,38]. The implementation details and derived feature files supporting this study are available from the corresponding author upon reasonable request.
